# Living in relationship with the Ocean to transform governance in the UN Ocean Decade

**DOI:** 10.1371/journal.pbio.3001828

**Published:** 2022-10-17

**Authors:** Michelle Bender, Rachel Bustamante, Kelsey Leonard

**Affiliations:** 1 Earth Law Center, Durango, Colorado, United States of America; 2 School of Environment, Resources, and Sustainability, University of Waterloo, Waterloo, Ontario, Canada; Brown University, UNITED STATES

## Abstract

Humanity’s relationship with the Ocean needs to be transformed to effectively address the multitude of governance crises facing the Ocean, including overfishing, climate change, pollution, and habitat destruction. Earth law, including Rights of Nature, provides a pathway to center humanity as a part of Nature and transform our relationship from one of dominion and separateness towards holism and mutual enhancement. Within the Earth law framework, an Ocean-centered approach views humanity as interconnected with the Ocean, recognizes societies’ collective duty and reciprocal responsibility to protect and conserve the Ocean, and puts aside short-term gain to respect and protect future generations of all life and the Ocean’s capacity to regenerate and sustain natural cycles. This Essay presents Ocean-centered governance as an approach to help achieve the 10 challenges for collective impact put forward as part of the UN Decade of Ocean Science for Sustainable Development and therefore living in a harmonious relationship with the Ocean.

## Abstract 2

Humanity’s relationship with the Ocean needs to be transformed to effectively address the many governance crises facing the Ocean. This Essay advances principles for Ocean-centered governance aimed to help meet the challenges of the UN Ocean Decade.

## Introduction

The UN Decade of Ocean Science for Sustainable Development (2021 to 2030) aims to transform Ocean science to support sustainable development, such as via Sustainable Development Goal (SDG) 14 (Life Below Water), and to connect people to the Ocean [1,2]. The UN General Assembly declared the UN Ocean Decade in December 2017 after the Intergovernmental Oceanographic Commission (IOC) of UNESCO developed a proposal for the decade to champion new oceanographic scientific, technological, and research advancements to support Ocean sustainability, as outlined in SDG14 [3,4]. The IOC was further charged with developing an implementation plan for the decade and established 7 societal outcomes and identified 10 decadal challenges (Table 1) [3].

**Table 1:** The UN Decade of Ocean Science for Sustainable Development 10 challenges for collective impact and 7 societal outcomes.

Challenge	Outcome
1. Understand and beat marine pollution	A clean Ocean where sources of pollution are identified and reduced or removed.
2. Protect and restore ecosystems and biodiversity	
3. Sustainably feed the global population	A healthy and resilient Ocean where marine ecosystems are understood, protected, restored, and managed.
4. Develop a sustainable and equitable Ocean economy	A productive Ocean supporting sustainable food supply and a sustainable Ocean economy.
5. Unlock Ocean-based solutions to climate change	A predicted Ocean where society understands and can respond to changing Ocean conditions.
6. Increase community resilience to Ocean hazards	A safe Ocean where life and livelihoods are protected from Ocean-related hazards.
7. Expand the Global Ocean Observing System	An accessible Ocean with open and equitable access to data, information and technology, and innovation.
8. Create a digital representation of the Ocean	An inspiring and engaging Ocean where society understands and values the Ocean in relation to human well-being and sustainable development.
9. Skills, knowledge, and technology for all	
10. Change humanity’s relationship with the Ocean	

Information in this table is from the UN Ocean Decade (https://www.oceandecade.org/).

Several global environmental principles and norms (such as sustainable and equitable use) have long been debated, ill-defined on the global scale and not yet effectively implemented [5,6]. Most importantly, the Ocean has historically been underrepresented within international regimes that implement these environmental principles. For example, the UN Framework Convention on Climate Change does not reference the UN Convention on the Law of the Sea (UNCLOS), and the Paris Agreement only mentions the Ocean in the preamble (therefore holding “less legal value than the Treaty”) and vaguely references the conservation of “sinks and reservoirs of greenhouse gasses” in Article 5, despite the Ocean’s vital role in regulating and dictating climate [7–10]. There is growing discourse calling for the development of a new Ocean ethos, of established and widely accepted norms, legal principles and collective values, for greater representation of the Ocean in international law, and recognition of the Ocean’s vital processes in regulating our climate and sustaining life beyond acting as a carbon sink [8,11].

In order to deliver the science needed for a “well-functioning ocean” and achieve SDG14, it is important to note that tension exists regarding the definition and guiding frameworks for “sustainability” [1,12]. Ensuring development meets present needs while not compromising the needs of future generations has been found to be far more difficult to achieve in practice, and the SDGs have been criticized for the way growth is measured, continued adherence to “business as usual” practices, economically focused metrics, the persistence of inequality and injustices, and the lack of a holistic view to not only achieve each goal, but also in acknowledging the relationships between goals [13–15]. As a result, many call for a stronger interpretation of sustainability that addresses the root causes, as well as the consequences, of environmental destruction and provides a diverse understanding of the value of Nature beyond instrumental and human-centered values [15,16]. As noted by Campagna and colleagues, “[c]hanging this not only requires complying with the scientific evidence of dependency of humanity on nature, but forces the conservation community to analyze its concept of nature and clarify the ethical grounds for valuing life” [17]. Maintaining the status quo of environmental law equates to the legalized destruction of Nature. International law needs to evolve to reflect the Ocean’s inherent rights to exist, flourish, and regenerate. Ocean health is human health.

In this Essay, we offer Earth law as a framework that can act as a catalyst to transform humanity’s relationship with Nature and ensure science and sustainable development expand beyond a utilitarian dimension [17]. Earth law is a philosophy of law based upon “the interdependence among humans and the environment” and guided by principles of holism, mutual enhancement, and reciprocal responsibilities, among others [16,18]. Earth law promotes a greater respect for all living things on Earth by recognizing that nonhumans have inherent rights and value, merely by existing [19,20]. This connection with Earth is restored vis-à-vis the holistic reconceptualization, adaptability, and flexibility of human ethics, institutions, and laws [16,19]. Rights of Nature is one legal framework within the body of Earth law. As evidenced by global comparative studies, Rights of Nature recognizes Nature as a living being with inherent rights and that society has a right to defend and protect Nature [16,18–22]. Therefore, the emerging Rights of Nature movement seeks to illustrate Nature as valued for itself (intrinsic value), no longer viewed as an object or property, but as a subject with rights [23]. As such, references to the Ocean, Ocean-centered governance, and Nature are capitalized in this Essay to be consistent with the Rights of Nature framing and recognition as a legal entity and noun. For example, Helen Dancer posits that this distinction, albeit contentious, frames “Nature as subject,” rather than as “service-provider,” as is commonly articulated in discourse of the human–Earth relationship, and has been embedded within global frameworks, particularly prevalent in Latin America (Constitution of Ecuador) and globally seen within the UN Harmony with Nature project [24]. Indeed, this assumption is not novel, but reflects histories of belief systems and worldviews; “the Ancient Greek Earth goddess, Gaia, Ancient Celtic belief systems in Europe and the cosmovisions of many Indigenous Peoples today center on respect for Mother Earth” [24]. Following the scholarship of Stefan Helmreich, we seek to invoke a higher respect for the Ocean and Nature, not as objects, but living entities. Helmreich emphasizes the need for all scientists to “renarrate,” “reorient,” and reposition our relationship to the Ocean through processes of “oceanization.” A process of justice that promotes Ocean vitality by affirming the Ocean as living and disrupting colonial webs of ecological subjugation embedded in social, political, and economic systems since the onset of the Anthropocene [25,26].

Earth law advances several assumptions in the conceptualization and implementation of ecocentric legal theory (or Earth-centered governance) and, therefore, the necessity to advance a new paradigm for Ocean governance. First, Earth law assumes that all of Nature (ecosystems and species, plants, microorganisms and animals, as well as biotic and abiotic components) have inherent and fundamental rights [17]. Earth law also assumes that humans exist as a part of Nature within an inextricable and complex web of relationships, and as a result, human rights are embedded within and dependent on the realization of Nature’s inherent rights [27–29]. Just as humans have rights based on our existence and being, so too does Nature. Second, to date, environmental law has largely contributed to, and is unable to effectively react to, the growing environmental crisis. This is predominantly the result of a colonial worldview of humanity as separate from and owners of Nature, largely anthropocentric (human-centered) environmental laws and policies, and equating Nature to a resource and property with value derived from benefit and utility to humankind [20,27,30–34]. Although not all worldviews and legal frameworks adopt this paradigm, within this Essay, we use the vision for the Ocean Decade: “the science we need for the ocean we want,” as an example of international community norms and values that convey that humans are the primary beneficiary of a healthy Ocean [1]. Earth-centered governance, on the other hand, represents a paradigm shift in law and in thinking about humans as a part of the Earth system, aiming to understand and respect “the interactions between living (human and nonhuman) and nonliving Earth system constituents and processes, the multiple intertwined and complex governance challenges arising from such interactions, and particularly the deepening interconnected social-ecological disruptions through a complex web of feedback loops” [35]. As a result, Earth law offers a new overarching framework, based upon a shared ethic that embeds humans within Nature, for which sustainability can be reinterpreted under, address many of the concerns with its implementation, and ensure effective realization of the UN Decade of Ocean Science societal outcomes.

We asked ourselves “how might Ocean-centered governance principles transform the types of solutions put forward to address the UN Ocean Decade Challenges?” In answering this question, this Essay explores how Earth law can reshape Ocean governance to prioritize the needs of the Ocean to address societal outcomes for Ocean well-being. The development of Earth-centered law is still in its infancy in its application for Ocean protection, but precedents exist to inform the development of how humanity may respect the inextricable relationship with the Ocean and ensure science and governance frameworks such as “sustainability” center Ocean needs properly within Ocean governance (i.e., Ocean-centered governance). In this Essay, we review each of the 10 Ocean Decade challenges of the UN Decade of Ocean Science for Sustainable Development to identify pathways for solutions to pressing Ocean crises following Ocean-centered governance principles. Across these Ocean challenges, the UN Ocean Decade developed 7 outcomes to describe the “Ocean we want” (Table 1). Building upon the existing literature, we highlight case studies of Ocean-centered governance across jurisdictional scales. Some examples are unique to the legal system, culture, issues, or politics present and the way they would apply or be implemented will therefore be dependent upon myriad factors including the geographic, temporal, and jurisdictional scales.

Additionally, we acknowledge that many Indigenous Peoples do not express their relationships with other humans and the natural world in terms of “rights” and that care is needed in considering appropriate ways to engage with law due to the role that western law has played in the colonization and subjugation of Indigenous Peoples, lands, and waters. As a result, tension exists between some applications of Rights of Nature and Indigenous worldviews and customary law, which has been found to overstate the connection between Rights of Nature and the legal framework being an Indigenous philosophy, as well as findings of co-opting Indigenous ontologies into universalist hegemonies [36–38]. However, we adopt a more conscientious approach of Earth law and Earth-centered governance, which offers a broader interpretation of Rights of Nature in order to address such concerns. Additionally, this provides a blueprint of concrete actions that scientists, practitioners, and world leaders can adopt to promote Ocean-centered governance to advance the UN Ocean Decade outcomes worldwide.

### Ocean-centered governance

**Fig 1:**
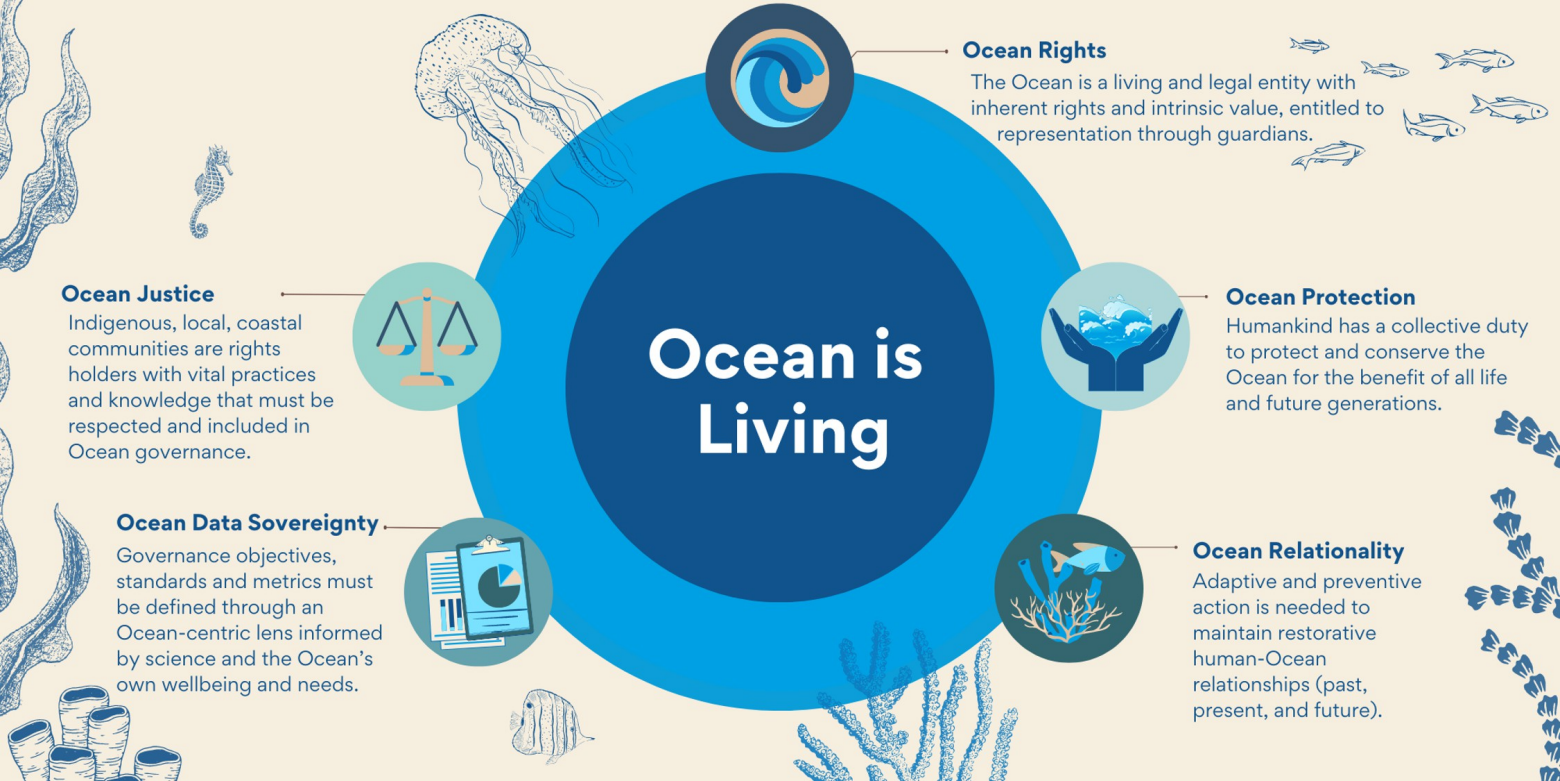
Ocean-centered principles to guide Ocean governance transformation. Interconnected relationships between Ocean-centered governance principles of justice, data sovereignty, rights, protection, and relationality rippling out from the key understanding that the Ocean is living. Transformation in Ocean governance requires action across all 5 principles. Created by Rachel Bustamante via Canva.com.

In order to achieve effective implementation of the UN Ocean Decade, we must recognize the interconnection and interdependence of humankind with the Ocean. One pathway to do so is through an Ocean-centered approach, which fundamentally is based upon governance principles that prioritize and apply the ecological needs and interests of the Ocean; in other words, a process that promotes scientists and decision-makers to shift from an anthropocentric lens to an Ocean-centered lens. Though great strides have been made since the industrial era, western governance systems have largely failed to protect the complex interactions and relationships between humankind and the Ocean, consider diverse knowledge systems, and include Indigenous, local, and coastal communities and traditional knowledge and science [6,39–41]. This disenfranchisement of scientific plurality maintains “business as usual” strategies rather than promoting shifts to imaginative and novel governance approaches to address our unbalanced relationship with the Ocean. In writing this Essay, we conceptualized 5 principles for Ocean-centered governance with the aim of developing more inclusive approaches for Ocean science and sustainability (Fig 1 and S1 Appendix).

Building upon Earth law and Rights of Nature understandings, Ocean-centered governance recognizes the Ocean as a living entity, advancing law, policy, and institutional action that centers the needs of the Ocean in decision-making. By positioning the Ocean as a living entity with inherent rights, governance advances understandings that the Ocean has agency, is an actor worthy of representation, and that democratization of global Ocean governance must be inclusive of Ocean values and diverse “waves of knowing” or deep ancestral knowledge and connections to place that center Ocean relationality [42,43]. As political scientist Karin Amimoto Ingersoll underscores, Indigenous Peoples and coastal communities have rich “seascape epistemologies” built on millennia of coexisting with and learning from the Ocean [42]. This line of thinking suggests that we need radical and revolutionary transformation in how we imagine ourselves within a collective Oceanic future [44,45]. Ocean-centered governance therefore embraces the plurality of Oceanic knowledge, culture, and identities and enables us to ask ourselves “what is the science the Ocean needs” for a shared harmonious future. Not only recognizing the rights of the Ocean, but also its intrinsic values, may transform the human–Ocean relationship and provide the paradigm shift necessary in order to restore Ocean health [46].

Ocean-centered governance seeks to create new legal mechanisms that act as a catalyst for humanity to rethink our role as an inherent part of the Oceanic system, recognize the Ocean as “an entity that maintains its existence and functions as a whole through the interaction of its parts,” and become responsive to and understands the functioning of the Ocean and “the entire community of life it hosts” and supports [47–50]. Therefore, an Ocean-centered approach also places human and economic activity within the natural capacity of the Ocean and adopts an integrated, holistic, systems, and life cycle approach [51].

Importantly, an Earth-centered approach to governance is not purely defined by rights [52]. Rights equate to a statement of societal values and create a new ethic for conservation [53]. Relational- and responsibility-based values in practice leads to the reinterpretation of, or strict adherence to, key governance principles while facilitating the development of ecologically based criteria and standards. For example, Panama’s National Rights of Nature law (Ley N° 287) includes the respect for Indigenous cosmology, the prevention principle, precautionary principle, higher interest of Nature, restoration, and *in dubio pro natura* (when in doubt err on the side of Nature) [54]. In addition to recognizing that Nature has rights to exist, persist, and regenerate vital cycles (among others), has intrinsic values outside human utility, and that every person has the right to demand respect for Nature’s rights, the law is to be governed by the identified principles. Representation is a key principle within an Ocean-centered approach [55]. Not only does the principle ensure stakeholder representation in decision-making, but also the representation of Nature, such as through “guardians” or “trustees.” Though trusteeship is not new (e.g., applications of the Public Trust Doctrine), guardians under a Rights of Nature framework are legally required to represent the intrinsic value and interests of Nature and put aside human interests [55–57]. Additionally, Nature’s interests and needs inform scientific standards determining sustainability. For example, scientists convened in California in 2015 to discuss how a definition of “a healthy ocean” may comply with the Ocean’s own interests and needs, outside human benefit and utility, and lead to more adaptive and preventive management of human impacts to Ocean health [58]. Though much more work is needed to develop Ocean-centered standards, centering the Ocean’s needs in decision-making is a core component of an Ocean-centered approach and is further expanded upon in this Essay.

In the following sections, we explore how an Ocean-centered approach can produce innovative and effective solutions to each UN Ocean decadal challenge. This discussion explores specific Ocean-centered examples of policy responses or legal interventions that we recognize have been uniquely and contextually applied in practice. These cases represent and help clarify how an Ocean-centered approach is applicable to addressing each challenge and what implementation might look like in practice.

### Challenge 1: Understand and beat marine pollution

Marine pollution showcases the inseparable and transboundary nature between land-based activities and Ocean health, highlighting the need to transform governance to address the relationship between democracy and sustainability, intergenerational justice, equity, and the distribution of environmental costs and benefits [59–62]. For example, plastic materials are cheaper to produce than renewable or biodegradable materials largely because pollution is not considered in the cost. This is due to our economic system being linear, focused on short-term gain and failing to adequately account for externalities [61].

Adopting an Ocean-centered lens to address marine pollution requires a life cycle approach to shape patterns of production and consumption and address pollution at the source (whether it be plastic production, agricultural runoff, dumping of mine tailings, etc.). Currently, the UN Environment Agency resolution “End Plastic Pollution: Towards a legally binding instrument” is entering negotiations on the global stage to address “the entire life cycle of plastics, from extraction of raw materials to legacy plastic pollution,” thereby transferring the cost of impacts on plastic to producers and signaling a market transition to circular products while incentivizing recovery and recycling [63–68].

Naturally, as reduction of pollutants to “near zero-input” in the Ocean necessitates collective action and coordinated governance in all respects, a holistic approach has been suggested to be most effective [63]. Thus, this represents an opportunity to frame the negotiations and development of the plastics treaty around an Ocean-centered approach, as it requires holistically assessing Ocean well-being interconnected to human activity, which can be fundamental to implementing regulations to halt marine pollution at the local, national, and international levels. Overall, an Ocean-centered approach to address pollution creates a shift in consumer values and producer practices by taking into account what the Ocean needs to be healthy. As such, cost-benefit analysis on plastic products would include externalities, impacts to human health and the health of other species and ecosystems, for present and future generations, from extraction to disposal [64].

The City of Santa Monica, California offers an example of how “public education and a local focus on shifting legal perspectives and consumer values can lead to larger cultural shifts” [69]. For example, the City passed a Sustainability Ordinance in 2013 recognizing “the rights of people, natural communities, and ecosystems to exist, regenerate, and flourish” [70]. This effort began first with a resolution, identifying a philosophical foundation and environmental ethic for human activity within the city, and the Ordinance was incorporated into the City’s Sustainability Plan. Guiding principles for the Plan include the recognition of the local communities’ linkage with the regional, national, and global community and the commitment towards sustainable rights for its residents, natural communities, and ecosystems [71]. Bans of plastic bags and nonmarine-degradable food service containers are examples of local actions taken by the city to mitigate plastic pollution. As scientist Max Liboiron highlights, anticolonial science, especially within the field of oceanography, acknowledges that plastic pollution is not merely a condition of market systems or reliance on fossil fuels but intertwined with colonialism and Indigenous land and water dispossession [61]. If the Ocean is recognized as a living entity with rights to be respected (Fig 1), then regulatory instruments would reorient the standards and metrics therein to holistically include ecologically based criteria to control pollution and dismantle colonial extractivism. This could apply in cases of scientific uncertainty, where a strengthened application of precaution could prove necessary. For example, the severity of toxicant effects of plastic and their interactions with other contaminants at varying concentrations, distributions and ecosystem conditions to harming marine life is still in early stages of understanding [68]. Overall, an Ocean-centered approach invokes the responsibility of all sectors to consider human interests but ultimately respect ecological and planetary boundaries.

### Challenge 2: Protect and restore ecosystems and biodiversity

Over the past 50 years, marine biodiversity has declined by 49% [72–74]. Predominantly, conservation interventions are reactive, taking effect once species and their ecosystems are threatened or endangered, and without addressing the primary drivers of biodiversity loss or the cumulative effects of human activity [24]. For governance practices to protect the Ocean (Fig 1), they must actually bring about “changes on or in the water” [59]. Ocean-centered governance aims to incorporate anticipatory, adaptive, and flexible decision-making by reconsidering core values and recognizing the intrinsic worth of the Ocean and constitutive species, ecosystems, biodiversity, and abiotic and biotic components [75]. Recognition of the intrinsic value of biodiversity is essential in order to effectively protect and restore ecosystems and ensure development is sustainable. Fortunately, changes in this direction can now be seen at all scales. For example, the current negotiations to develop a new treaty under UNCLOS to protect biodiversity on the High Seas include the stewardship principle and the obligation to “preserve the inherent value of biodiversity of areas beyond national jurisdiction (draft text as of IGC5)” [76]. Additionally, the Convention on Biological Diversity preamble recognizes State’s to be “[c]onscious of the intrinsic value of biological diversity” [77]. This would facilitate changes in how decision makers currently decide what level of human activity is “sustainable” and what may constitute “severe or irreversible harm” in international waters, which to date, have been overexploited [78].

However, no international standard currently exists for the inclusion of intrinsic worth of biodiversity in decision-making. Local examples exist, such as in Aotearoa/New Zealand, where Māori successfully negotiated the Te Awa Tupua (Whanganui River Claims Settlement) Act of 2017 around the intrinsic values of the River or Tupua te Kawa. Those values provide that “Te Awa Tupua is a living and indivisible whole…[including] all of its physical and metaphysical elements,” “the source of spiritual and physical sustenance”, and “sustains both the life and natural resources within the Whanganui River and the health and well-being of the iwi, hapū, and other communities of the River” [57]. As a result, the Act requires that any person exercising a function under another identified law must recognize and have regard to not only the legal status of the River, but also its intrinsic values [57]. The Te Awa Tupua Act explicitly defines diverse values and relationships surrounding the River and local communities. This example highlights how governance, and therefore an Ocean-centered approach, can begin to integrate social, economic, and environmental impacts from both a monetary and nonmonetary view of the existence of biodiversity [75].

The Intergovernmental Science Policy Platform on Biodiversity and Ecosystem Services (IPBES) Values Assessment illuminates that the causes of and solutions for our global environmental challenges are tightly linked to the ways in which we value our environments [79]. Importantly, the report concludes that environmental policy is “more likely to foster transformative change” when aligned with and incorporating “the diverse values of nature,” recognizing Rights of Nature can advance both justice and sustainability by addressing the diverse ways in which people relate to and value Nature [79]. In fact, the IPBES produced a methodological assessment that calls for the inclusion of worldviews, broad values (moral principles), and specific values, including intrinsic values, suggesting that local examples such as Te Awa Tupua are vital and should be incorporated into assessments and implementation of environmental policy [79].

Beyond the inclusion of the intrinsic value of Nature, the recognition of Nature’s inherent rights enables proactive and adaptive management in order to protect, defend, and restore Nature’s rights. For example, the Special Law of the Galapagos for Ecuador’s Galapagos Marine Reserve states that citizens and Nature are both guaranteed the constitutional right of living well (adhering to the 2008 Constitutional Amendment recognizing Nature as having inherent rights) [80]. In fact a guiding principle for governance is to create an equilibrium among society, the economy, and Nature. As a result, industrial fishing and harming sharks in the archipelago were entirely prohibited to protect sharks and maintain the ecosystem under “minimal human interference” [80]. With this example, we can see how policy proactively protected sharks in the Reserve, and adapted to scientific evidence on the importance of sharks as keystone species, and to the local economy via ecotourism [81]. Similarly, principles in Ecuador’s environmental code and Panama’s National Law are vital to an Ocean-centered approach, including *in dubio pro natura* (when in doubt favor Nature) and the prevention principle, to encourage decision-making to err on the side of Nature in any case of doubt regarding the impacts of human activity. In order to ensure ecosystems and biodiversity are protected and restored, it is critical that governments apply proactive and adaptive measures. Recognition of the Ocean’s legal agency and intrinsic value provide a mechanism to legally require such measures [54,82].

### Challenge 3: Sustainably feed the global population

Policies to regulate fishing as well as harmful fishing subsidies highlight the “unprecedented power that humans exert over nonhuman Nature” and the inequalities in allocation, agency, and justice (including the disproportionate burden on Indigenous Peoples, developing States, big Ocean States, and local communities) [83,84]. Valuing fish as a resource, such as objectifying a population as a “fishery,” and using maximum sustainable yield (MSY) fails to fully grasp the needs of the system as whole, and the nonhuman species within it, as well as adequately accounting for future impacts, including climate change [85]. For example, the European Union’s Common Fisheries Policy and total allowable catch (based upon MSY) was found to be “on average 48% higher than those advised by scientists” [86,87]. As a result, MSY, as a metric to regulate human behavior, allows governance to focus on short-term needs rather than maintaining a healthy and thriving ecosystem for future generations of all species. Though enshrined in international governance (UNCLOS Article 61), more holistic measures continue to be explored that are inclusive of whole-ecosystem processes and dynamics along with social and economic factors, such as reframing MSY as a limit, rather than a target, or managing human activity with higher metrics of precaution, rather than total allowable catches [88–90].

An Ocean-centered approach is a valuable lens from which to reevaluate human relationships with fish populations (Fig 1), and is arguably, a research need moving forward. As noted above, Ocean-centered governance ensures the agency or representation of nonhuman stakeholders in decision-making processes, such as through human guardians (other terms used include protectors, stewards, trustees, and custodians) and recognizes the global population as including all species, with humans as just one entity within the system, thereby constraining economic activity within ecological limits [55,91]. For example, ʔEsdilagh First Nation Sturgeon River Law of 2020 states “[p]eople, animals, fish, plants, the nen (“lands”), and the tu (“waters”) have rights in the decisions about their care and use that must be considered and respected” [92]. It further calls for proactive planning and management to ensure the health of the tu is maintained, the consideration of the needs of the fish, plants, and other relations before taking from or using tu and allows for the ʔEsdilagh Government to suspend or cancel an authorization where necessary to protect fish, habitat, and water flow [93]. In 2020, the Tsilhqot’in Nation similarly voiced concerns that stronger action was needed in order to mitigate extinction risk of Fraser River Chinook salmon, and even forfeited their fishing rights in order to preserve salmon for future generations [93]. Ensuring the agency and representation of marine biodiversity in decision-making processes addresses a major challenge in democratization, supports a shift in power to those communities most affected by poor governance, and ensures a fair and equitable process to feed the global population by ascribing the responsibility on humankind to ensure intergenerational equity. Therefore, Ocean-centered governance empowers adaptive and preventive action to maintain restorative human–Ocean relationships.

### Challenge 4: Develop a sustainable and equitable Ocean economy

Current global frameworks promoting sustainable development inherently focus on human rights and needs and hold “anthropocentric notions of equity” (e.g., principle 1 of the Rio Declaration states “human beings are at the center of concerns for sustainable development”) [46,94]. In addition to recognizing the agency of the Ocean, we can propel a mutually enhancing and equitable economy by redefining law and policy concepts to embrace principles of justice for the Ocean and people [95]. Ocean justice (Fig 1) supports the inherent rights of the Ocean as a living entity worthy of protection expanding notions of marine jurisdiction to be inclusive of diverse actors including the Ocean itself [95]. A rethink of sustainability is necessary going forward, and could take the form of “ecological sustainability,” defined as: “the maintenance of life support systems and the achievement of a ‘natural’ extinction rate” [96]. In practice, our existing institutions (economy, governance, laws, etc.) and policymaking tools (cost-benefit analysis, qualitative data, etc.) can shift to the recognition that the “economy is a subsystem of human society which is a subsystem of the Earth” [97]. Doing so will help to fulfill the standards of intergenerational equity, a future healthy and livable planet for future generations of humans and all life on Earth [98]. A sustainable Ocean economy is guided by and adaptive to Ocean health, respective and responsive to ecological boundaries. For example, “sumak kawsay,” “suma qamaña,” “küme Mongen,” “buen vivir,” or “good living,” originating from Andean Indigenous ontologies (such as Quechua, Aymara, and Mapuche communities, respectively), identifies well-being and development as “community-centric, ecologically balanced, and culturally sensitive” [99]. These concepts “both reflect a fundamental morality (respect for ecological integrity) and require action [to protect and restore]” Nature while sustaining human activity [100].

Importantly, more work is needed to include and amplify the agency of Indigenous and local communities, customary law, science, and other “waves of knowing” in decision-making processes, providing genuine participation in governance [42]. Fundamentally, an inclusive process will not appropriate knowledge, but will deconstruct colonial ideologies, seeking to redress historical exclusion, dispossession and disregard of Indigenous sovereign rights and Ocean-relations [39,101,102]. It is critical we not only ensure economic activity is consistent with respecting the ecological integrity of the Ocean, but also that the ecological integrity is defined outside human benefit and utility. An equitable Ocean economy must ensure Indigenous, local, and coastal communities are rights holders that are respected and included in Ocean governance.

Another concept to reimagine is the western-derived understanding of “what is healthy.” Maintaining a “healthy ocean” constitutes a main objective of laws and policies worldwide [58,103]. However, this standard and similar metrics to measure whether activities support health are largely based upon human benefit and utility, viewing benefits as “ecosystem services” rather than a holistic view of the benefits a healthy Ocean provides to other species, ecosystems, functions, and systems [104,105]. For example, Ruhl and Salzman position that ecosystems have long been valued “as a source of valuable commodities and recreational pursuits that, obviously, do not always align with the goal of maintaining ecological integrity” [106]. Linda Sheehan postulates a definition of health as “normal form and function” over a long period of time and “demonstrat[ing] sufficient organization, vigor, and resilience to allow ecosystems and species to exist, thrive, and evolve as natural systems within the context of their expected natural life spans” [58]. Ocean-centered governance recognizes Oceanic ecosystems as all intrinsically valuable and determines metrics for what constitutes a “healthy” Ocean as defined by the Ocean’s intrinsic needs, including chemical, physical, and biological needs, rather than the Ocean’s utility as a human resource or economic benefit [58,107]. These metrics and valuations of the Ocean are then integrated and fundamental to economic policy decisions, guiding development towards a circular and reciprocal Ocean economy.

### Challenge 5: Unlock Ocean-based solutions to climate change

Climate scientists have highlighted that existing adaptation strategies to climate change often overlook systemic injustices and promote colonialism, environmental racism, and anthropocentric ideologies while international frameworks are siloed from each other and are only beginning to recognize and respect the Ocean–climate nexus [102,108,109]. In fact, the World Meteorological Organization “revealed that 4 key climate indicators broke new records in 2021: sea-level rise; ocean heat; ocean acidification; and greenhouse gas concentrations” [110]. Ocean-centered governance provides an opportunity to unlock Ocean-based solutions to climate change grounded in principles of relationality, interconnectivity, and equity, whereby the rights and needs of the Ocean are prioritized for climate action alongside other relations [62,111]. Globally, mitigation solutions and adaptation frameworks need to be designed to fit local contexts and the specific needs of the Ocean and coastal communities disproportionately impacted. For example, the WAMPUM Adaptation Framework is an Ocean-centric approach to sea-level rise adaptation planning, focusing on first observing ecosystem change indicators, identifying traditional ecological knowledge solutions, and then restoring wetlands, seagrass, aquatic species, and other life-giving relatives to support Ocean well-being [102].

Moreover, Ocean-centered solutions would recognize the inherent rights of blue carbon ecosystems as living entities. For example, the government of Belize recognized the Belize Barrier Reef as a living entity in 2011 [112,113]. In 2009, a ship ran aground causing extensive damage to the reef and the Government of Belize brought legal action against the shipowners, arguing the reef was not “property” but rather a living entity and that they were its “custodian and guardian” [113]. The government was awarded damages beyond those of liability to property requiring the shipowners to pay not only for physical damage, but also the reparation costs of the loss of habitat, protection against erosion and storm surge, and biodiversity as well as the monetary restitution for damage caused to tourism, recreational, aesthetic, and cultural value [113]. Ocean-centered governance promotes transformation towards a harmonized human–Ocean relationship by being reflexive and adaptive to the needs and acknowledging the agency of the Ocean and blue carbon ecosystems, recognizing the interconnectivity of all life among changing climatic conditions.

### Challenge 6: Increase community resilience to Ocean hazards

Blue carbon ecosystems not only provide Ocean-based climate solutions, but also they provide early warning and hazard reduction services for coastal communities in the face of climate emergency [114]. Ocean-centered governance builds adaptive and preventative measures to extreme climate events. Coastal hazards are often exacerbated by human exploitation and overdevelopment of the coastline [115]. Community preparedness and resilience will require innovative advancements in law to address compounding climate change hazards across geographies and scales. As an example, there is a growing surfing movement to protect the Ocean, coastlines, and, in particular, Waves, from overdevelopment and land use degradation using innovative Ocean-centric legal mechanisms [116]. In 2016, the Chicama wave, considered to be the longest left-breaking wave in the world, was granted legal protections under Peruvian national law. The law prohibits changes to the coastline and seabed that would alter the integrity of the wave [117,118]. The surfing community is a critical stakeholder in promoting community resilience and Ocean conservation [118–120].

Similarly, in 2020 the state of Victoria, Australia recognized the Great Ocean Road as “one living and integrated natural entity” under section 1(a) of the Great Ocean Road and Environs Protection Act [121]. These measures were taken to protect the region from infrastructure sprawl and ensure environmentally sustainable development, particularly noting the urgent need to holistically mitigate current and projected climate impacts affecting the coastlines. Moreover, the Act acknowledges the “intrinsic connection” of Indigenous Peoples to the sea and affirms their responsibility as caretakers and decision-makers [122]. These approaches highlight how Ocean-centered governance can reverse perspectives of the Ocean as hazard and reframe human–Ocean relationships as interdependent. Coastal hazard managers often base decisions on the application of cost-benefit analyses that commodify ecological and cultural benefits, but exclude long-term planning and ecological needs of shorelines. For example, as these assessments cannot fully quantify the human–Ocean relationship, Revell and colleagues suggest application of a precautionary approach [123]. An Ocean-centered approach applies ecological-based criteria in risk assessment and evaluates Ocean health needs now and based upon modeled future projections when deciding whether there is scientific uncertainty of potential harm from coastal development or other human activities. Finally, an Ocean-centered approach could help ensure inclusion and representation of Indigenous, local, and coastal communities with active voices and roles in coastal decision-making and planning, amplifying stewardship practices which have been known and integral to many of their communities since time immemorial [41,124].

### Challenge 7: Expand the Global Ocean Observing System

Existing Ocean observing systems prioritize human needs over the Ocean’s for observation, data collection, and priority setting [125–130]. “Restorying” Ocean observing system architecture to prioritize and embrace the science the Ocean needs is critical to address persistent ecological changes and crises. According to political scientist Jeff Corntassel (Cherokee Nation), restorying is the process by which institutions reexamine the dominance of colonial histories and erasure of Indigenous Peoples in stories of place [131]. Restorying information systems for Ocean observing is critical to democratizing data ecosystems to be inclusive of diverse peoples and “waves of knowing” [42]. This could be realized through ensuring enhanced monitoring efforts to better track biological data and best practices for Ocean conservation [130,132] (Fig 1). Data collection processes can also build in frameworks that support Indigenous Data Sovereignty [133]. The Ira Moana Project based in Aotearoa/New Zealand has incorporated principles of Indigenous Data Sovereignty to retain Indigenous provenance information of marine genetic samples in metadata of large marine databases to protect the rights of Indigenous Peoples [134,135]. These policies have broad applicability for ethical applications in future environmental DNA sampling in marine environments. Future data collection standardization efforts may also provide for data governance to be entrusted to Ocean guardians charged with protecting the Ocean’s needs. Monitoring should be inclusive of legal and policy changes that recognize Ocean ecosystems as living entities and that existing and emerging marine life programs adopt preventative measures to combat monetization of Ocean data. For example, essential ocean variables (EOVs) are integral measurements to understand the connection between the Ocean and Earth’s climate system, focusing “on the physics of the ocean system, the biogeochemistry, and the biology and ecosystems” [136,137]. EOVs and essential climate variables (ECVs) provide critical information to assess the health of the Ocean and enable predictions of climate change impacts and associated adaptation needs [136,137].

If global Ocean observation systems recognize the inherent rights of the Ocean and marine life, this would engage a more integrated and interconnected approach, alleviating several known challenges of sustaining these systems in a collaborative environment [138,139]. Révelard and colleagues confirm that all stakeholders of the Ocean observing system “need to establish a shared vision and commit to common priorities,” addressing the current “absence of a well-established overall governance framework” [139]. The lack of sustained long-term funding associated with Ocean observing systems, and associated costs in facilitating an integrated approach is one foreseen challenge that would benefit from future research. An observing system that centers the Ocean’s well-being in data collection and analysis, establishes a collective view of the Ocean as living, and seeks to realize a harmonized human–Ocean relationship can help enhance protection of Oceanic ecosystems. A key component of recognizing the Ocean as a rightsholder, is ensuring representation and ownership of data is protected across the totality of the data ecosystem (all the data components, models, and infrastructure) and life cycle (from collection to analytics and governance). Going forward, governance of the Global Ocean Observing System could include Ocean guardians as steering committee members or as a new expert panel that can act as a voice for the Ocean and advance EOVs with the greatest potential for the protection of the Ocean’s right to exist, flourish, and naturally evolve. Enhancing Ocean observation systems [140] will also provide valuable validation metrics on the efficacy of Rights of Nature instruments in protecting Ocean and coastal habitats. Without legislative, policy, and legal drivers, observing systems will miss critical opportunities for innovation in Ocean protection [130,141].

Ocean data sovereignty also encompasses Indigenous data governance principles, including the CARE Principles: collective benefit, authority to control, responsibility, and ethics [142]. The CARE principles provide standards to measure operationalization of ethical systems architecture in support of the Ocean’s data needs. Moreover, these principles ensure equitable distribution of benefits from current and future data use. However, steps towards implementation must ensure data collection, use, and governance protect individual and collective rights of Indigenous Peoples, local and coastal communities, and the Ocean. Ocean observing systems must democratize to not only be more inclusive of diverse knowledge systems but also center the data needs of the Ocean for holistic health in perpetuity.

### Challenge 8: Create a digital representation of the Ocean

Reliance on Mercator projections to digitally represent the Ocean has led to centering on western positionality [143]. Drawing on scholarship of legal geography, Ntona and Schröder invite us “to question how […] representational devices (e.g., maps) and information management technologies (e.g., geographic information systems) associated with [Ocean mapping] work together to “gentrify” marine spaces, constructing them in ways that reflect the hierarchy of values and the differentiated rights of access” [144]. The Ocean is not a static entity but a living and “lively space,” and digital representations must account for the fluid plurality of existence and material manifestations [144]. Ocean-centered governance recognizes the Ocean as a data actor and rightsholder.

Digital representations of the Ocean, including the development of a dynamic Ocean map, must center the Ocean uninterrupted by imagined state borders and Exclusive Economic Zones [145]. Epeli Hau’ofa advocates for society to rethink colonial logics of the sea as divisive and rather embrace that “we’re connected rather than separated by the sea” [146]. Charne Lavery further argues such projections would debunk continental centricity to focus on “oceanicity,” “indicating the degree to which a place is overall subject to the influence of the sea” [143]. Therefore, Ocean-centered governance emphasizes principles that empower oceanicity to radically transform digital representations of the Ocean so that the Ocean itself is the center of focus, rather than geopolitical borders of terrestrial occupation.

As Kira Coley highlights, Ocean mapping is critical to building resilience for the Ocean and the global community requiring commitments to accessibility and diverse stakeholders to be co-creators of mapping initiatives [147]. The Spilhaus World Ocean Map is often used as an example of an Ocean-centric map, but as the author notes “a map of the world ocean is essentially a world map” because Earth is an Ocean planet [145,148]. However, it has been decades since these Ocean maps were created and a new digital Ocean map is needed that fully reflects the dynamism and stories of diverse waves of knowing Ocean relationality. Connecting people to the Ocean through innovative digital representations (e.g., maps) that propel oceanicity and orient users to points of centricity of greatest importance to the Ocean can have lasting transformative impact on science, law, and policy for the Ocean Decade [149].

### Challenge 9: Skills, knowledge, and technology for all

The knowledge, practices, and technology that embody and advance an Ocean-centered approach must be made accessible to all Ocean users [150–152]. Recent developments in literature have sought to quantify global Rights of Nature initiatives and Earth law governance reports, analyses, laws, and policy advancements, such as a mapping analysis by Kauffman and colleagues, and as expanded upon by Putzer and colleagues, whose database has over 400 logged Rights of Nature initiatives, spanning across 39 countries (S1 Appendix) [52]. Ocean-specific repositories of data and best practices, such as the IOC-UNESCO Ocean Best Practices system, can elevate living in harmony with Nature and crosslink to other existing repositories.

To ensure comprehensive knowledge and technology across all aspects of Ocean science, Ocean governance must also carve out space for valuing Indigenous knowledge, traditions, and management [41,146]. Historically, Indigenous nations and communities have been widely excluded, underrepresented, and culturally erased in international forums and Ocean policy. Intentional work is needed to ensure genuine participation and agency along with deconstructing resounding colonial legacies that dispossess Indigenous communities from Ocean spaces [153]. For example, the Marae Moana Act, a conservation act of the Cook Islands that, among other purposes, develops a marine spatial plan and designates Marine Protected Areas, includes space for Cook Islanders’ traditional knowledge “around marine custodianship including ra’ui and ra’ui mutukore” [154]. Ra’ui is a temporary ban on the extraction of a species and ra’ui mutukore is a permanent ban, both of which are determined by a tribal chief, and are utilized “to enhance food security, intrinsic value, protect and improve biodiversity, rehabilitated or restored areas and traditional customary practices” [155].

Several global frameworks have recognized the importance of ensuring Indigenous rights to self-determination, including the Convention on Biological Diversity Aichi Targets and UN Declaration on the Rights of Indigenous Peoples. However, Indigenous scholars Fischer and colleagues, conclude that implementation and enforcement of these standards is uneven [31]. Equitable decision-making requires more than involvement, but representation in conservation science, and respect of Indigenous rights and leadership. These practices are most recommended contextually “for Indigenous Peoples whose conception of rights is that it comes with coinciding responsibilities to steward or care for the environment” [98]. Bridging diverse scientific traditions including Indigenous knowledge and science can help us to better understand and sustain the interconnected relationship we have with the Ocean and lead to more effective protection of Ocean health [31]. By ensuring the best science and technology is available and used by all, and bridging diverse scientific traditions, the global community can advance innovation and adaptation, as well as address power imbalances and allocation inequalities [84].

### Challenge 10: Change humanity’s relationship with the Ocean

The Ocean faces imminent danger of losing its capacity to support life, including from increased human-induced pressures such as climate change, overfishing, and land-based pollution [8]. The persisting “heavily privatised, zoned, and securitised Ocean undermines the human-Ocean relationship” and has led to a focus on rights to exploit over responsibilities to protect and preserve [40,90]. In order for the Ocean to continue to support life, “a new relationship between humanity and the Ocean is required” [8,39,40,51].

Ocean scientists and practitioners argue a biocultural framework would provide a transition to enhanced human–Ocean reciprocal relationality [156]. A biocultural approach is evident in many Indigenous and “place-based coastal communities” and emphasizes the “mutual interdependence of biological and cultural diversity into complementary, relational values of humans with one another and with nature” [156]. Situated within this biocultural perspective, the Ocean is a living being worthy of care and healing, as a whole. A “more-than-human-world” is a key concept in this perspective [8,156]. Across Oceania, governance principles of stewardship or guardianship are prevalent and help guide many communities to live in a harmonious relationship with the Ocean [8,31,40,145,157,158]. For example, in Aotearoa/New Zealand, laws and policies include kaitiakitanga, which is “the exercise of guardianship by the tangata whenua of an area in accordance with tikanga Māori in relation to natural and physical resources” and emphasizes the human responsibility to nurture and care for the environment [158,159]. The values of kaitiakitanga are relational and are considered a principle of law that predates common law [159]; cases in point include the Resource Management Act and the Sea Change–Tai Timu Tai Pari Hauraki Gulf Marine Spatial Plan (the Plan). The Plan has 4 fundamental pillars for governance and states that considering the Gulf “as a being in its own right will help us to rethink our reciprocal responsibilities and work toward a better balance” [160]. Additionally, the Plan recognizes the Māori and local Iwi as spiritual guardians or protectors (kaitiaki) of the lands, territories, and waters they have ancestrally possessed, having a personal and collective responsibility to maintain them. In practice, this includes setting temporary restrictions on fishing within certain areas, using the lunar calendar to guide planting and harvesting, banning recreational fishing and birding, harvesting only what is needed, and Iwi feedback and consent on regulations and permits within the Continental Shelf [161,162].

Kaitiakitanga was recently discussed heavily in a Supreme Court decision in New Zealand regarding whether consents should be given to undertake seabed mining. In addition to ruling that if the environment cannot be protected from harm through regulation then the activity must be prohibited, the court ruled that the decision-making committee needed to consider existing interests, which included the kaitiakitanga of iwi. The impact on spiritual and cultural values was underestimated and seabed mining was found to be inconsistent with the iwi parties’ exercise of kaitiakitanga to protect the life force of the marine environment [160].

Kaitiakitanga and other Indigenous values and understandings are important for decision makers to engage with, recognize and respect, as they can help transform humanity’s relationship with the Ocean by establishing a shared common vision and goals, and “creating principled guiding frameworks and processes to facilitate coherent systems-oriented regulations” [8]. Moreover, recognizing that humankind has a collective duty (reciprocal responsibilities) to protect and conserve the Ocean for the benefit of all life and future generations may maintain mutually beneficial and restorative human–Ocean relationships [55].

## Conclusion

In this Essay, we explored examples of Earth law internationally, how governance principles and standards are defined and applied in practice, and how they can inform the 10 decadal challenges. Recognizing the Ocean as a living being is increasingly important for planetary well-being and global sustainability. We have presented Ocean-centered governance principles and examples to guide implementation to help transform the vision of the Ocean Decade towards supporting the science needed for the Ocean that the Ocean wants. To ensure full and effective realization of the 10 decadal challenges (Table 1), and therefore the sustainable use of the Ocean, humankind must transform our relationship with the Ocean and evolve our perceptions and values guiding the challenges (Box 1). Although the 10th challenge itself calls for this transformation, this challenge must be completed first and foremost and guide the implementation of the 9 other challenges. Shifting our relationship with the Ocean from one of ownership and separateness towards loving interdependence, reciprocity, and reverence can transform Ocean governance.

**Box 1. Five Ocean-centric principles to transform Ocean governance**

**Ocean rights**

Recognizing the Ocean as a living being, stakeholder, and rightsholder and hearing the Ocean’s voice in decision-making can positively influence every challenge to Ocean health by reorienting standards for decision-making and governance principles to respect the Ocean’s rights to life, to restoration, and to flourish.

**Ocean relationality**

Restoring a balanced and reciprocal relationship with the Ocean leads to a greater fulfillment of human rights in tandem; grounding human activity based upon this interconnection will enhance respect of ecological/planetary boundaries and help ensure resilience for all life while respecting and amplifying, but not appropriating, Indigenous and local knowledge from communities that have long known and practiced these relations in Ocean spaces.

**Ocean data sovereignty**

Global observing systems can create imaginative and accessible technological infrastructure, orienting digital users to points of Ocean centricity that address fragmentation in normative, technical, temporal, spatial, and scalar interactions, as well as alleviate power inequalities.

**Ocean protection**

A shared and collective responsibility of all to protect and conserve the Ocean can shift the imbalances between allocation and use, and a life cycle approach can shape patterns of production and consumption to advance holistic Ocean regeneration.

**Ocean justice**

Recognizing the Ocean’s agency encourages democratization, supports a shift in power to those communities most affected by poor governance, and ensures a fair and equitable process to nurture the global population by ascribing the responsibility on humankind to ensure intergenerational equity.

The UN Ocean Decade scientific community is uniquely positioned to advance wise practices for living in relationship with the Ocean. Adopting an Ocean-centered governance approach is vital to inform the development of how humanity may respect the inextricable relationship with the Ocean and ensure science and governance frameworks, such as those determining “sustainability” can meet the Ocean Decade challenges. Centering the Ocean as living, with humanity as part of the Oceanic system, can produce not only the science we need for the Ocean we want, but also the science the Ocean needs and wants.

## Supporting information

S1 Appendix Summary of materials and methods. File documenting materials and methods used for identifying Ocean rights laws and initiatives for Ocean-centered governance.
(DOCX)

## Abbreviations:

ECV: essential climate variable
EOV: essential ocean variable
IPBES: Intergovernmental Science Policy Platform on Biodiversity and Ecosystem Services
IOC: Intergovernmental Oceanographic Commission
MSY: maximum sustainable yield
SDG: Sustainable Development Goal
UNCLOS: UN Convention on the Law of the Sea
