# Supplementary material for: Living in relationship with the Ocean to transform governance in the UN Ocean Decade
Source: PLoS Biol. 2022 Oct 17;20(10):e3001828. doi: 10.1371/journal.pbio.3001828 (PMC9576050; doi:10.1371/journal.pbio.3001828)
Supplement: S1 Appendix — File documenting materials and methods used for identifying Ocean rights laws and initiatives for Ocean-centered governance. (DOCX) [file pbio.3001828.s001.docx]

S1 Appendix

**Materials and Methods**

The principles of an Ocean-centered approach presented in this Essay result from a review of Earth Laws, specifically a subset of legal mechanisms categorized as Rights of Nature laws, policies and judicial decisions, and a literature review and desktop analysis of the ten challenges to collective action identified by the UN Decade of Ocean Science for Sustainable Development. We asked ourselves “how might Ocean-centered governance principles transform the types of solutions put forward to address the UN Ocean Decade Challenges?”

**Review of Existing Rights of Nature Databases**

Within several countries are examples of Rights of Nature initiatives in the form of constitutional amendments, national law, judicial decisions, treaty agreements, Indigenous law, or local law (e.g., Ecuador, Panama, Mexico, Bolivia, India, Colombia, Brazil, New Zealand, Yurok Tribe, and the United States) [1]. A repository of Rights of Nature laws and policies exist within several platforms, namely the United Nations Harmony with Nature Initiative Platform [2]. Noting limitations of this data, the authors likewise, consulted newly developed datasets by the Global Alliance for the Rights of Nature (GARN) and affiliated academics Kauffman and Putzer.^^[[1]](#footnote-0)^^ The author team reviewed both datasets, and found that of hundreds of laws documented to date less than 10% specifically reference ocean or coastal ecosystems or species [1, 3]. Though all general Rights of Nature laws include all parts of Nature, including the Ocean, examples where specific ecosystems have gained ‘legal personhood/entity’ status or otherwise, are largely limited to land-based ecosystems.

**Select Rights of Nature Laws with Principles for Earth-centric Governance**

Additional analysis of existing Rights of Nature laws (i.e. those with Earth-centered principles) and those laws with specific Ocean/coastal references were reviewed and informed the development of our Ocean-centered approach. These laws include both those successfully passed or introduced in legislative sessions (See Table A).

**Table A.** Rights of Nature Laws with Earth-Centered Governance Principles

| **Law** | **Year** | **Location** |
| --- | --- | --- |
| Ecuador Rights of Nature Constitutional Amendment | 2008 | Ecuador [4] |
| Organic Environmental Code | 2017 | Ecuador [5] |
| Panama National Rights of Nature Law | 2022 | Panama [6] |
| Bolivia Law 071 | 2010 | Bolivia [7] |
| ʔElhdaqox Dechen Ts’edilhtan (ʔEsdilagh Sturgeon River Law) | 2020 | Esdilagh First Nation/ Canada [8] |
| Magpie River Resolution 025-21 | 2021 | Innu First Nation/Canada [9] |
| Defense and Recognition of Rivers Rights and other Water Sources of the State of Oaxaca | 2020 | Mexico [10] |
| New Zealand Te Awa Tupua Act | 2017 | Iwi Whanganui/ Aotearoa/ New Zealand [11] |
| Mar Menor (proposed law) | 2022 | Spain [12] |
| Uganda National Environment Act | 2019 | Uganda [13] |
| Santa Monica Sustainability Rights Ordinance | 2014 | Santa Monica, USA [14] |
| Wadden Sea (proposed law) | 2019 | Netherlands [15] |
| Loyalty Islands, New Caledonia Environmental Code | 2016 | Loyalty Islands, New Caledonia [16] |

The author team then analyzed the existing principles guiding international Ocean governance. We note that laws of the sea have evolved over the last century, including positive and transformative language changes in international governance. For example, the 1958 Geneva Convention on Fishing and the Conservation of Living Resources of the High Seas, designated marine conservation as “rendering possible the optimum sustainable yield from those resources so as to secure a maximum supply of food and other marine products,” [17, Art. 2, p. 2] whereas the 1982 Convention requires states to “protect and preserve the marine environment” [18, p. 100, 19). However, “freedom of the High Seas” remains a tangential provision to states’ obligation to conserve (18, Art. 87, p. 57). Though not an “absolute norm,” it remains a contentious point of debate in discussions of the UN Treaty for Marine Biodiversity Beyond National Jurisdiction [20]. Freestone [19] identifies these principles along with several others: “Conservation of high seas living marine resources and biodiversity; Sustainable and equitable use; Cooperation; Precautionary Approach (including prior EIA); Ecosystem approach; Use of best available science; Transparency; Responsibility of states to control the actions of their nationals and consequences for breach of international legal obligations” (19, p. 385). Despite Freestone’s observations, the author does not identify Rights of Nature or Earth Jurisprudence in any form. Neither does he identify intergenerational equity and Indigenous rights, which are widely part of Ocean governance discourse and inherent to good governance.

**Scoping Review - Peer Review Literature Gaps**

Not only does a gap exist in applying Rights of Nature within ocean law and policy, but literature gaps also exist. Ocean-centered governance is not a new concept for many communities globally, especially Indigenous, local, and coastal communities who have fostered interdependent relationships with the Ocean for millenia [21, 22, 23, 24] activity within foundational framings of connection and responsibility [25, 26, 27]. However, these Ocean-centered governance approaches are rarely cited in Ocean governance literature. Franke et al. calls for the “integration of human health and well-being, environmental ethics, ocean governance, and the natural and social sciences in a more encompassing framework of ocean health” [28, p. 558]. The author team conducted a scoping review of peer reviewed articles addressing theoretical considerations for recognizing the inherent rights of the Ocean as a living entity. Utilizing Scopus, Google Scholar, and Web of Science only 3 articles were identified that specifically mention “Rights of Nature” and “Ocean” in their title, abstract, or keywords (See Table B) [29, 30, 31].

**Table B.** Search Criteria used to identify Ocean-centered governance concepts

| **Criterion** | **Details** |
| --- | --- |
| Search Terms | “Ocean” AND “Rights of Nature” |
| Language | English |
| Timeframe | Up to May 1, 2022 |
| Databases | Scopus, Web of Science, Google Scholar |
| Inclusion criteria | Primarily related to and describing characteristics of Ocean Rights as protected by Rights of Nature. |

In 2017, the Earth Law Center launched the Ocean Rights Program to facilitate the introduction of Rights of Nature framing in Ocean governance, and has developed a multitude of resources to do so, including The Earth Law Framework for Marine Protected Areas, which highlighted eleven guiding principles to guide marine protected area practitioners towards an Ocean-centered approach [32]. Studies have since explored how Rights of Nature frameworks could be applied to international ocean governance [33], including the Marine Biodiversity of Areas Beyond National Jurisdiction (BBNJ) Agreement [29, 34].

**Scoping Review - News Articles**

Despite this area of study being largely neglected in Ocean science peer-reviewed literature, searches of news articles conducted in May 2022 in Factive and Nexis Uni found 88 and 230 article results identifying Ocean rights respectively as of May 1, 2022. This may highlight a disconnect between the grassroot movements for Ocean governance approaches emerging globally documented by journalists in media and those being promoted by scientists in academic journals on the UN Ocean Decade. Scholars have previously noted that not all communities and peoples have access to decision-making within UN institutions including Indigenous Peoples [35].

**Word Cloud Analysis - UN Ocean Decade Challenges**

The author team utilized the text of the UN Ocean Decade Challenges to create a word cloud that analyzes frequency patterns of words within the text to create an illustration based on word occurrence [36,37]. The word cloud was then used to assess thematic trends [38]. Five themes emerged from the text of the challenges including: values, relationship, protect, data, and equitable (See Figure A).

**Figure A.** Word cloud Five Emergent Themes from UN Ocean Decade Ten Challenges for Collective Impact


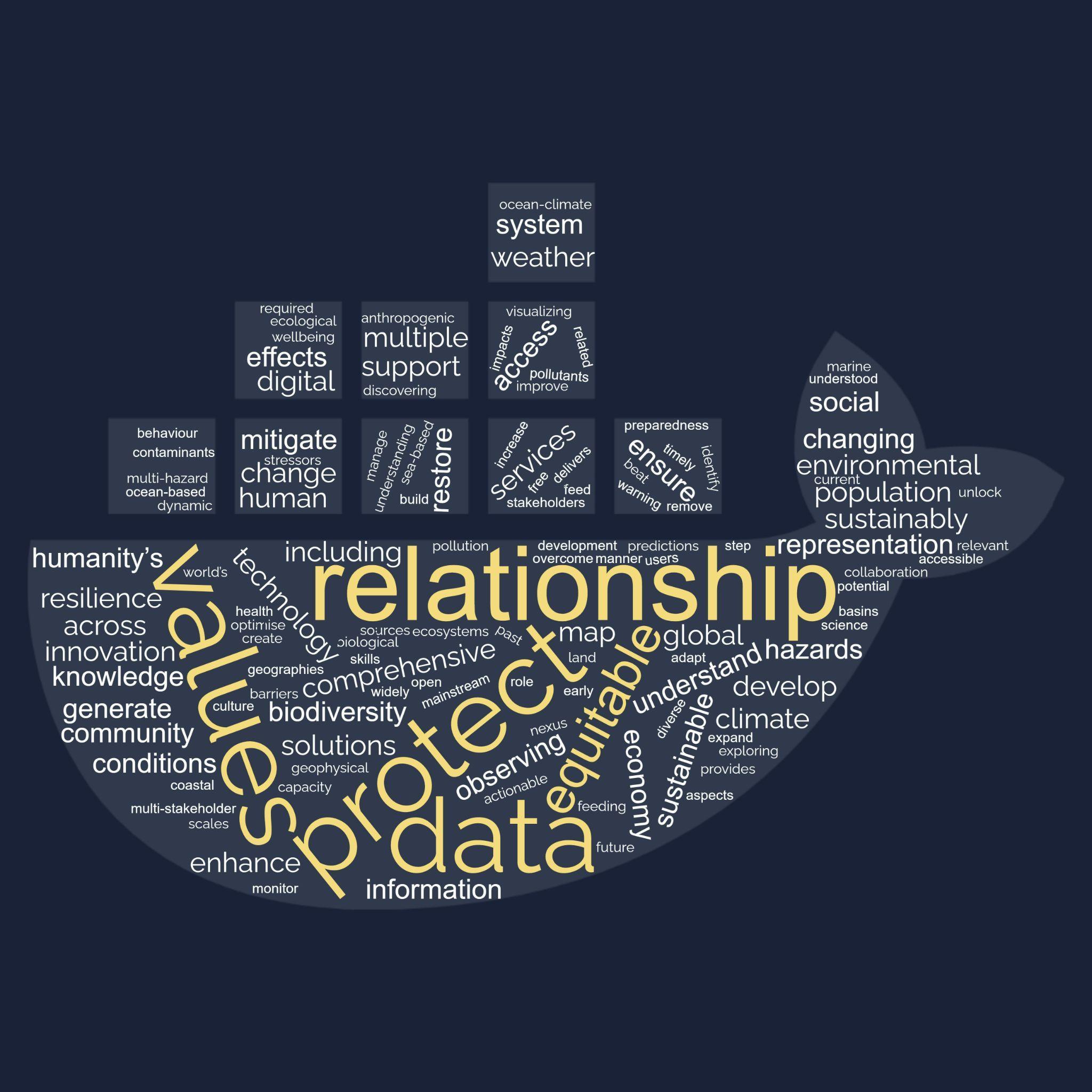


Although these themes were present in the challenge text the governance metrics were not defined through an Ocean-centric lens. Our team was then tasked with identifying how might these themes best support Ocean-centered governance approaches. To assess this we used these themes to develop search criteria for a desktop review of peer reviewed literature to identify Ocean-centered governance principles (See Table C).

**Table C.** Search Criteria used to identify content for Ocean-centered governance principles

| **Criterion** | **Details** |
| --- | --- |
| Equitable - Search Terms | “Ocean Equitable” OR “Ocean Equity” OR “Ocean Justice” OR “Blue Justice” |
| Relationship - Search Terms | “Ocean Rights” OR “Ocean Relationality” OR “Ocean Relationship” OR “Ocean Relation*” |
| Values - Search Terms | “Ocean Value*” |
| Data - Search Terms | “Ocean Data” OR “Ocean AND Data Sovereignty” OR “Ocean Observing” |
| Protect - Search Terms | “Ocean Protect” OR “Ocean Protection” |
| Language | English |
| Timeframe | Up to May 1, 2022 |
| Databases | Scopus, Web of Science, Google Scholar |
| Inclusion criteria | Primarily related to and describing characteristics of Ocean and/or marine governance |

The Essay draws on literature found in this search to identify Ocean-centered governance principles across the ten UN Ocean Decade challenges.

**References**

[1] Kauffman CM, Martin PL. Constructing Rights of Nature Norms in the US, Ecuador, and New Zealand. Global Environmental Politics [Internet]. 2018 Nov [cited 2022 Aug 23];18(4):43–62. Available from: <https://direct.mit.edu/glep/article/18/4/43-62/15016>

[2] United Nations. Harmony With Nature [Internet]. Available from: <http://www.harmonywithnatureun.org/>

[3] Putzer A, Lambooy T, Jeurissen R, Kim E. Putting the rights of nature on the map. A quantitative analysis of rights of nature initiatives across the world. Journal of Maps [Internet]. 2022 Jun 13 [cited 2022 Aug 23];1–8. Available from: <https://www.tandfonline.com/doi/full/10.1080/17445647.2022.2079432>

[4] “Ecuador Rights of Nature Constitutional Amendment”. 2008. United Nations. Harmony With Nature [Internet]. Available from: <http://files.harmonywithnatureun.org/uploads/upload657.pdf>

[5] Ecuador. Organic Code on the Environment [Internet]. 2017. Available from: <https://www.climate-laws.org/geographies/ecuador/laws/organic-code-on-the-environment>

[6] La Asamblea Nacional de la Republica de Panama. Ley 287. Que reconoce los derechos de la Naturaleza y las obligaciones del Estado relacionadas con estos derechos. 2022.

[7] Bolivia Law 071. [Internet]. 2010. Available from: <http://www.worldfuturefund.org/Projects/Indicators/motherearthbolivia.html>

[8] Esdilagh First Nation Chief and Council. ʔELHDAQOX DECHEN TS’EDILHTAN [Internet]. May, 2020. Available from: <http://files.harmonywithnatureun.org/uploads/upload995.pdf>.

[9] Magpie River Resolution 025-21. [Internet]. 2021. Available from: <http://files.harmonywithnatureun.org/uploads/upload1070.pdf>

[10] Defense and Recognition of Rivers Rights and other Water Sources of the State of Oaxaca. [Internet]. 2020. Available from: <http://files.harmonywithnatureun.org/uploads/upload1052.pdf>

[11] Te Awa Tupua (Whanganui River Claims Settlement) Act 2017, Public Act 2017 No 7 [Internet]. Mar 20, 2017. Available from: <http://www.legislation.govt.nz/act/public/2017/0007/latest/whole.html>

[12] Mar Menor. [Internet]. 2022. Available from: <http://files.harmonywithnatureun.org/uploads/upload1197.pdf>

[13] Uganda National Environmental Act. [Internet]. 2019. Available from: <http://files.harmonywithnatureun.org/uploads/upload834.pdf>

[14] City of Santa Monica. Chapter 12.02 SUSTAINABILITY RIGHTS [Internet]. 2013. Available from: <https://www.qcode.us/codes/santamonica/>

[15] Bijzondere rechten UNESCO Werelderfgoed Waddenzee (Wadden Sea). [Internet]. 2019. Available from: <http://files.harmonywithnatureun.org/uploads/upload974.pdf>

[16] CODE DE L’ENVIRONNEMENT DE LA PROVINCE DES ILES LOYAUTE. [Internet]. 2016. Available from: <http://files.harmonywithnatureun.org/uploads/upload704.pdf>

[17] Convention on Fishing and Conservation of the Living Resources of the High Seas [Internet]. United Nations Treaty Series; 1958. Available from: <https://treaties.un.org/pages/ViewDetails.aspx?src=TREATY&mtdsg_no=XXI-3&chapter=21>

[18] United Nations Treaty Series. Convention on the Law of the Sea. Dec 10, 1982. Art. 193 and Art. 192.

[19] Freestone D. Principles Applicable to Modern Oceans Governance. Int J Mar Coast Law [Internet]. 2008 [cited 2022 Aug 23];23(3):385–91. Available from: <https://brill.com/view/journals/estu/23/3/article-p385_1.xml>

[20] Young M. Then and Now: Reappraising Freedom of the Seas in Modern Law of the Sea. Ocean Development & International Law [Internet]. 2016 Apr 2 [cited 2022 Aug 23];47(2):165–85. Available from: <https://www.tandfonline.com/doi/full/10.1080/00908320.2016.1159088>

[21] Ingersoll KA. Waves of Knowing: A Seascape Epistemology [Internet]. Duke University Press; 2016 [cited 2022 Aug 23]. Available from: <https://www.degruyter.com/document/doi/10.1515/9780822373803/html>

[22] George RY, Wiebe SM. Fluid Decolonial Futures: Water as a Life, Ocean Citizenship and Seascape Relationality. New Political Science [Internet]. 2020 Oct 1 [cited 2022 Aug 23];42(4):498–520. Available from: <https://www.tandfonline.com/doi/full/10.1080/07393148.2020.1842706>

[23] Poe MR, Levin P. Looking Forward. In: Conservation for the Anthropocene Ocean [Internet]. Elsevier; 2017 [cited 2022 Aug 23]. p. 481–90. Available from: <https://linkinghub.elsevier.com/retrieve/pii/B9780128053751000234>

[24] Varanasi U, Trainer VL, Schumacker EJ. Taking the Long View for Oceans and Human Health Connection through Community Driven Science. IJERPH [Internet]. 2021 Mar 6 [cited 2022 Aug 23];18(5):2662. Available from: <https://www.mdpi.com/1660-4601/18/5/2662>

For example, many communities view the Ocean as a living sacred entity, and manage human

[25] van Uitregt V, Sullivan I, Watene K, Wehi P. Negotiating greater Māori participation in Antarctic and Southern Ocean research, policy, and governance. The Polar Journal [Internet]. 2022 Jan 2 [cited 2022 Aug 23];12(1):42–61. Available from: <https://www.tandfonline.com/doi/full/10.1080/2154896X.2022.2058222>

[26] Fischer M, Maxwell K, Nuunoq, Pedersen H, Greeno D, Jingwas N, et al. Empowering her guardians to nurture our Ocean’s future. Rev Fish Biol Fisheries [Internet]. 2022 Mar [cited 2022 Aug 23];32(1):271–96. Available from: <https://link.springer.com/10.1007/s11160-021-09679-3>

[27] Epeli Hau’ofa. Our Sea of Islands. In: Wilson R, Dirlik A, editors. Asia/Pacific as Space of Cultural Production [Internet]. Duke University Press; 2020 [cited 2022 Aug 23]. p. 86–98. Available from: <https://www.degruyter.com/document/doi/10.1515/9780822396116-008/html>

[28] Franke A, Blenckner T, Duarte CM, Ott K, Fleming LE, Antia A, et al. Operationalizing Ocean Health: Toward Integrated Research on Ocean Health and Recovery to Achieve Ocean Sustainability. One Earth [Internet]. 2020 Jun [cited 2022 Aug 23];2(6):557–65. Available from: <https://linkinghub.elsevier.com/retrieve/pii/S2590332220302499>

[29] Harden-Davies H, Humphries F, Maloney M, Wright G, Gjerde K, Vierros M. Rights of nature: perspectives for global ocean stewardship. Marine Policy. 2020 Dec 1;122:104059.

[30] Fitz-Henry E. Conjuring the past: Slow violence and the temporalities of environmental rights tribunals. Geoforum. 2020 Jan 1;108:259-66.

[31] Ponce VM, Oñate-Valdivieso F, Cobos-Aguilar R. The right of nature to dispose of its salts. Tecnología y ciencias del agua. 2018 Jun;9(3):1-28.

[32] Michelle Bender. The Earth Law Framework for Marine Protected Areas. [Internet]. Earth Law Center; 2018. Available from: <https://static1.squarespace.com/static/55914fd1e4b01fb0b851a814/t/5adca14b352f538288f4ea67/1524408668126/Final+Draft+3.pdf.>

[33] De Lucia V. Ocean commons, law of the sea and rights for the sea. Canadian Journal of Law & Jurisprudence. 2019 Feb; 32(1):45-57.

[34] Claudet J, Amon DJ, Blasiak R. Transformational opportunities for an equitable ocean commons. Proceedings of the National Academy of Sciences. 2021 Oct 19;118(42):e2117033118.

[35] Morgan R. Transforming law and institution: Indigenous peoples, the United Nations and human rights. Routledge; 2016 Feb 24.

[36] Ahuja V, Shakeel M. Twitter presence of jet airways-deriving customer insights using netnography and wordclouds. Procedia computer science. 2017 Jan 1;122:17-24.

[37] Kabir AI, Ahmed K, Karim R. Word Cloud and Sentiment Analysis of Amazon Earphones Reviews with R Programming Language. Informatica Economica. 2020 Oct 1;24(4):55-71.

[38] Ocean Decade [Internet]. 2021. Available from: <https://www.oceandecade.org/vision-mission/>

1. See Kauffman, Craig, Shrishtee Bajpai, Kelsey Leonard, Elizabeth McPherson, Pamela Martin, Alessandro Pelizzon, and Alex Putzer. Eco Jurisprudence Tracker. V1. 2022. Distributed by the Eco Jurisprudence Monitor.<https://ecojurisprudence.org/dashboard>. [↑](#footnote-ref-0)
